# Unraveling the genomic epidemiology and plasmid-mediated carbapenem resistance of *Klebsiella pasteurii*

**DOI:** 10.3389/fmicb.2025.1561624

**Published:** 2025-03-17

**Authors:** Xinyue Li, Zexuan Song, Jinshuo Liu, Jingguang Jin, Hanxia Wan, Huimin Chen, Xinhua Luo

**Affiliations:** ^1^National Institute for Communicable Disease Control and Prevention, Chinese Center for Disease Control and Prevention, Beijing, China; ^2^The First Clinical College, Wenzhou Medical University, Wenzhou, China; ^3^Department of Clinical Laboratory Medicine, Taizhou Municipal Hospital, Taizhou, China

**Keywords:** *Klebsiella pasteurii*, carbapenem resistance, plasmid, *bla*_KPC_, mobile genetic element, tilimycin and tilivalline

## Abstract

This study isolated a *Klebsiella pasteurii* strain, K1134, from the sputum of an ICU patient, revealing its resistance to the carbapenem antibiotics meropenem and imipenem. Whole-genome sequencing identified a plasmid pK1134-KPC, which carries the carbapenem resistance gene *bla*_KPC-2_. pK1134-KPC, belonging to the IncFII_pCP020359_ plasmid group, exhibits a modular structure with *bla*_KPC-2_ embedded in a 32.09 kb accessory region containing multiple accessory genetic elements (AGEs). Comparative genomic analysis of 48 *K. pasteurii* isolates from 12 countries showed high genetic diversity, with strains clustered into three clades. Notably, *K. pasteurii* harbors extensive antimicrobial resistance genes across diverse AGEs, classifying it as multidrug-resistant. Twelve *bla*_KPC_-carrying AGEs were identified from the sequences of the isolates, classified into two groups: Tn*7551* and Tn*6296*-related elements. The gene clusters for enterotoxins tilimycin and tilivalline, encompassing key regulators and operons, were present in nearly all strains, with incomplete clusters exclusively observed in clade 3 isolates. This study underscores the global dissemination and genetic adaptability of *K. pasteurii*, highlighting its potential role as a reservoir for resistance genes and emphasizing the need for robust surveillance to mitigate its public health impact.

## Introduction

The *Klebsiella oxytoca* species complex (KoSC) is part of the human microbiome (Wan et al., 2023), but also an opportunistic pathogen causing various infections, including antibiotic-associated hemorrhagic colitis (AAHC) (Yang et al., 2022; Long et al., 2022). Members of KoSC are highly versatile pathogens, isolated from a variety of infections including wound, intra-abdominal, urinary, and lower respiratory tract infections. Outbreaks often stem from environmental sources, and the subsequent spread of phylogroup members within healthcare settings presents a significant challenge—particularly in neonatal intensive care units (Cosic et al., 2021). The KoSC has been found to comprise nine species, and each species has been assigned based on average nucleotide identity (ANI) and *in silico* DNA–DNA hybridization (isDDH). *K. pasteurii* is a genospecies of KoSC defined by genomic, phenotypic and proteomic characteristics (Merla et al., 2019). Until now, this potential emerging pathogen has been reported in clinical settings in many countries. However, there is still a lack of comprehensive and large-scale genomic investigation to reveal the genetic heterogeneity in *K. pasteurii*.

The carbapenems, the latest generation of β-lactam antibiotics, are commonly used to treat infections that are resistant to nearly all other antibiotics, including those resistant to extended-spectrum β-lactams (Ma et al., 2021). In the Ambler classification, proteins responsible for carbapenem resistance are categorized into several classes: class A (KPC and certain GES variants), class D (OXA enzymes), and class B (metallo-β-lactamases, MBL) (Nordmann et al., 2012). A study of carbapenem-resistant KoSC strains collected from several hospitals in China showed that *K. pasteurii* strains have been found to carry *bla*_KPC_, *bla*_NDM_ and *bla*_IMP_ (Wan et al., 2023). One study indicated that *Klebsiella* sp. was the most common carbapenem-resistant Enterobacteriaceae (CRE) in samples isolated from patients (Stefaniak et al., 2024), and the most common carbapenem resistance gene (CRG) was *bla*_KPC_, including subtypes *bla*_KPC-2_ and *bla*_KPC-3_. In addition, another analysis of the KoSC genome showed that *bla*_KPC-2_ was the most prevalent acquired resistance gene (Long et al., 2022).

CRGs are usually located on plasmids or transposons and spread rapidly among bacteria by horizontal gene transfer (HGT), such as conjugation, transformation, or transduction (Botelho et al., 2019). Plasmids carry multiple drug resistance genes, often leading to multiple drug resistance. Currently, the most commonly used plasmid typing scheme is called Inc/rep typing. This classification method largely aligns with the conjugation-based scheme (Rozwandowicz et al., 2018). Plasmid-mediated transmission of CRGs among KoSC has been widely reported, such as the IncA plasmid carrying *bla*_VIM_ (Biedrzycka et al., 2023), the IncX3 plasmid carrying *bla*_NDM-1_ (Jiang et al., 2023), the IncHI2 plasmid carrying *bla*_SIM-1_ (Li et al., 2022). In addition, there are still many sequenced CRG-carrying plasmids from KoSC that could not be assigned to any known Inc groups.

The dissemination of KoSC in healthcare settings, coupled with its ability to colonize the skin, respiratory tract, and urogenital tract, as well as to invade the bloodstream, drives outbreaks of both community-acquired and nosocomial infections globally (Glabonjat et al., 2021). KoSC causes AAHC, which is linked to the ability of these strains to produce enterotoxins, namely tilimycin (TV) and tilivalline (TM), and these cytotoxins also contribute to colonization resistance (Osbelt et al., 2024). In an animal model, *K. oxytoca* carry a secondary metabolite biosynthetic gene cluster that encodes a nonribosomal peptide assembly pathway (Schneditz et al., 2014), and it comprises 12 genes.

Here, we reported a carbapenem drug resistant *K. pasteurii* isolate K1134 harboring carbapenemase-encoding gene *bla*_KPC-2_, and its genetic characteristics were comprehensively analyzed. Together with the 47 *K. pasteurii* genomes available in GenBank, we used a total collection of the 48 global *K. pasteurii* sequenced isolates for further genomic epidemiology analyses, aiming to reveal the population structure, disclose the prevalence of antimicrobial resistance and virulence, and dissect the genetic environment of *bla*_KPC_ in *K. pasteurii*, contributing to better understanding and management of its role in antimicrobial resistance and public health threats.

## Materials and methods

### Isolation, identification and antimicrobial susceptibility testing

Strain K1134 was isolated in 2020 from the sputum specimen of a patient from a tertiary hospital located in Beijing, China. Activity of Ambler class A/B/D carbapenemases in bacterial cell extracts was determined by a modified CarbaNP test (Feng et al., 2016). The presence of the *bla*_KPC_ gene was investigated using PCR assays. Bacterial species identification was done using genome sequence-based ANI analysis (Jain et al., 2018). Bacterial antimicrobial susceptibility was tested by BioMérieux VITEK 2, and interpreted as per the 2020 Clinical and Laboratory Standards Institute (CLSI) guidelines (Humphries et al., 2021).

### Genomic DNA extraction, sequencing, and sequence assembly

Bacterial genomic DNA was isolated using the UltraClean Microbial Kit (Qiagen, NW, Germany) and sequenced from a paired-end library with an average insert size of 350 bp (range: 150–600 bp) on a HiSeq sequencer (Illumina, CA, United States), as well as a shared DNA library with an average size of 15 kb (range: 10–20 kb) on a PacBio RSII sequencer (Pacific Biosciences, CA, United States). The paired-end short Illumina reads were used to correct the long PacBio reads with the software proovread (Hackl et al., 2014), and then corrected PacBio reads were assembled *de novo* using SMARTdenovo.^1^ The sequencing data were checked using NanoPack29 and FastQC.^2^

### Sequence annotation and bioinformatics

Open reading frames (ORFs) and pseudogenes were predicted using RAST 2.0^3^ combined with BLASTP/BLASTN searches against the UniProtKB/Swiss-Prot database^4^ and the RefSeq database.^5^ Annotation of resistance genes, mobile elements, and other features were carried out using the online databases including CARD,^6^ ResFinder,^7^ ISfinder,^8^ INTEGRALL^9^ and Tn Number Registry.^10^ Multiple and pairwise sequence comparisons were performed using MUSCLE 3.8.31 and BLASTN, respectively. Gene organization diagrams were drawn in Inkscape 1.0.^11^ Heatmaps were plotted with MeV 4.9.0.

### Phylogenetic analysis

All the publicly available genomes of *K. pasteurii* isolates in GenBank (*n* = 47, updated on September 9, 2024) were downloaded. The pairwise ANI values of the 48 global *K. pasteurii* genomes were calculated using FastANI. The *K. pasteurii* genome sequences were aligned to the complete chromosome sequence (GenBank accession number NZ_CP073236) of the reference strain Sb-24, and the core single nucleotide polymorphisms (SNPs) were identified by Mummer v3.2.^12^ All the SNPs in the repetitive DNA regions were identified and filtered by RepeatMasker.^13^ Based on the final recombination-free core SNPs, a maximum-likelihood phylogenetic tree was constructed using RAxML with a bootstrap iteration of 1,000 and displayed using ChiPlot (Xie et al., 2023).

## Results

### General descriptions

A *K. pasteurii* strain named K1134, was isolated from the sputum specimen of a male patient admitted to the ICU surgery unit during routine sampling and culturing. Antimicrobial susceptibility testing revealed that strain K1134 was resistant to the carbapenem drugs meropenem and imipenem (Table 1). Whole-genome sequencing of K1134 demonstrated the presence of a ~ 6.2 Mb circular chromosome and six plasmids (Table 2). These plasmids were identified as pK1134-KPC, pK1134-aadA, pK1134-qnrS, pK1134-NR1, pK1134-NR2, and pK1134-NR3. Comparative genomic analysis showed that strain K1134 shared an ANI of 99.55% with the reference *K. pasteurii* strain Sb-24, highlighting its close genetic relationship.

**Table 1:** Antimicrobial drug susceptibility profiles of *K. pasteurii* K1134.

Antibiotics	MIC values (μg/mL)	Antimicrobial susceptibility
Ceftazidime	32	R
Ticarcillin/clavulanic acid	≥128	R
Piperacilin/tazobactam	≥128	R
Aztreonam	≥64	R
Imipenem	≥16	R
Meropenem	≥16	R
Amikacin	≤2	S
Tobramycin	2	S
Ciprofloxacin	≥4	R
Levofloxacin	≥8	R
Tigecycline	≤0.5	S
Doxycycline	≥16	R
Minocycline	8	I
Trimethoprim/sulfamethoxazole	≥320	R

S, sensitive; I, intermediate; R, resistant.

**Table 2:** Whole genome information of *K. pasteurii* K1134.

Sequence	Mean G + C content (%)	Length (bp)	Total number of ORFs	MLST	Inc type	Accession number
cK1134	55.2%	6,245,792	6,043	ST499	—	CP163256
pK1134-KPC	53.0%	137,213	163	—	IncFII	CP163257
pK1134-aadA	51.5%	170,890	200	—	Untypable	CP163261
pK1134-qnrS	50.9%	48,497	76	—	IncN1	CP163262
pK1134-NR1	34.5%	40,827	61	—	Untypable	CP163258
pK1134-NR2	36.5%	4,372	6	—	Untypable	CP163259
pK1134-NR3	31.9%	2,909	—	—	Untypable	CP163260

—, not available.

### A *bla*_KPC-2_-carrying plasmid pK1134-KPC

**Figure 1:**
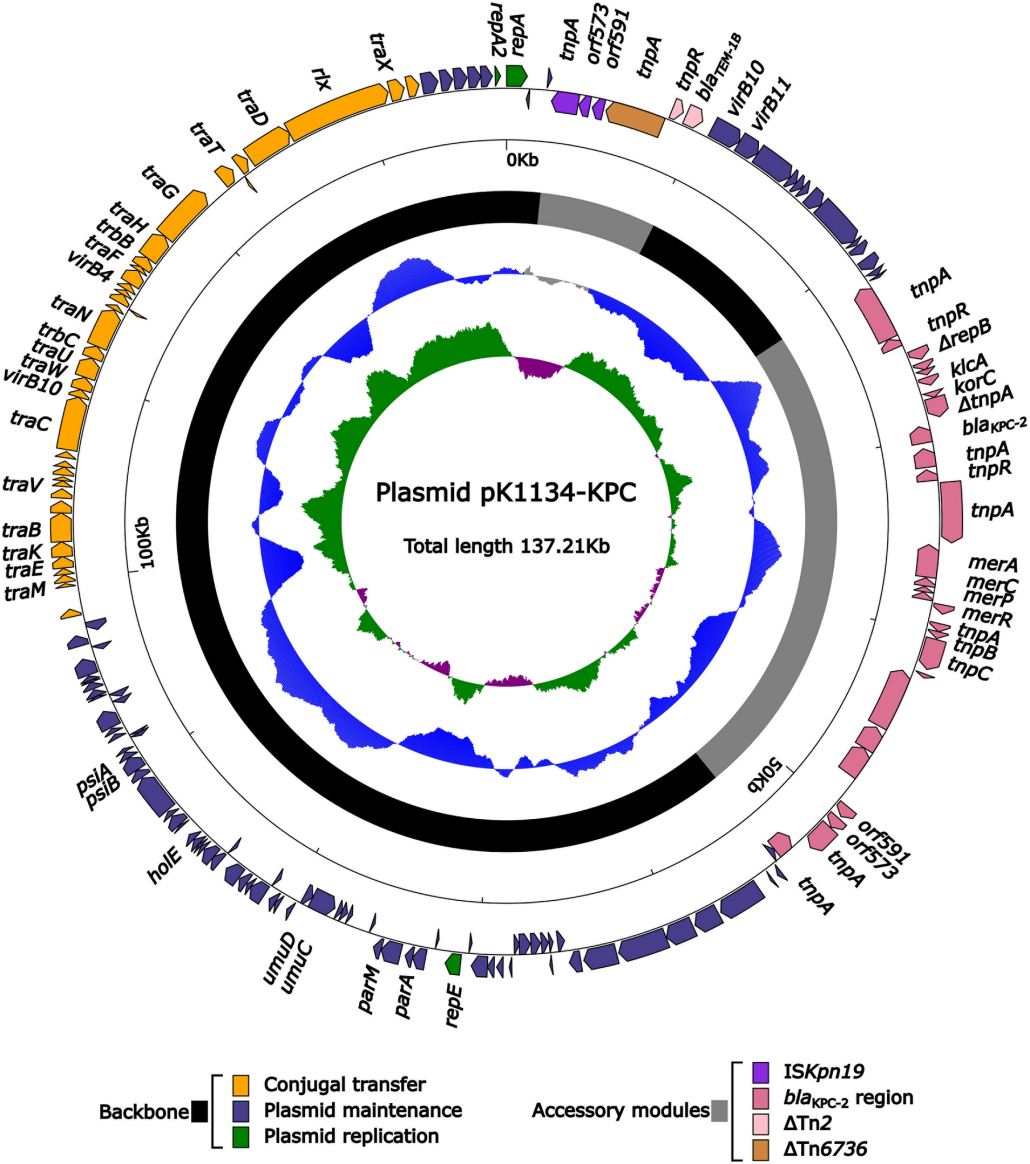
Schematic maps of the sequenced plasmid pK1134-KPC. Genes are denoted by arrows, and the backbone and accessory module regions are highlighted in black and gray, respectively. The next-to-innermost circle presents GC content.

**Figure 2:**
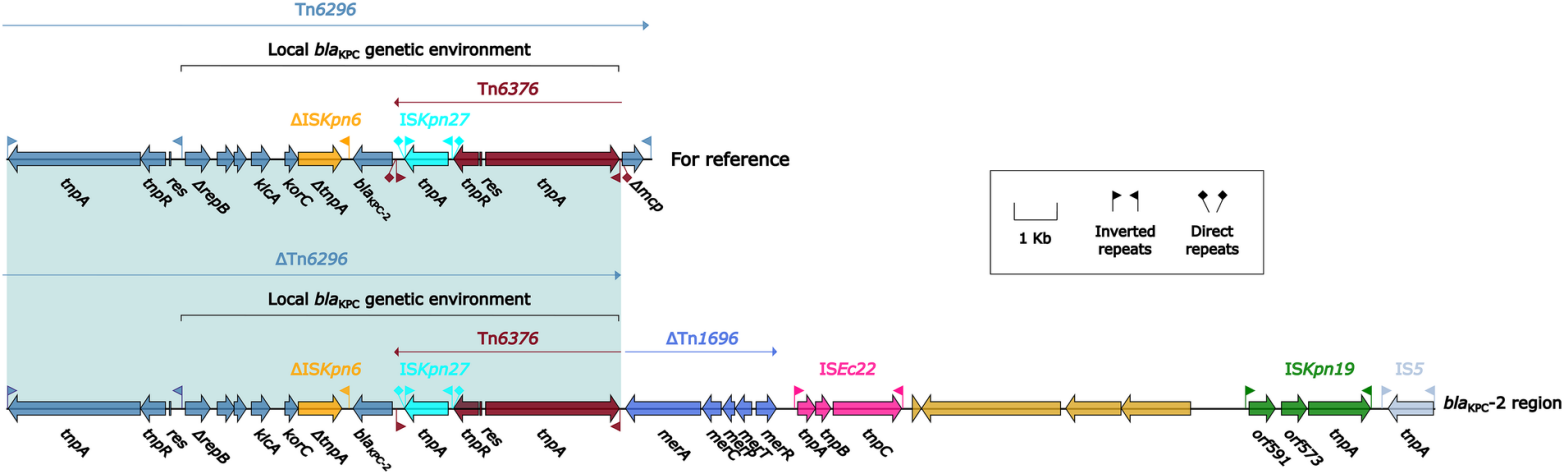
Organization of *bla*_KPC-2_ region and comparison to related regions. Genes are denoted by arrows. Genes, mobile elements and other features are colored based on their functional classification. Shading denotes regions of homology (nucleotide identity >95%). The accession number of Tn*6296* used as reference is FJ628167.

We identified one *bla*_KPC-2_-carrying plasmid pK1134-KPC from the isolate we collected, which had a circular DNA sequence of 137.21 kb in length, with an average GC content of 52.98%. The plasmid (Figure 1) was identified as belonging to the IncFII_pCP020359_ group because it and the reference plasmid unitig_2 (accession number CP020359) had identical *repA*_IncpCP020359_ genes sharing ≥95% nucleotide identity. The plasmid unitig_2 was found in the *K. oxytoca* strain AR_0147 in 2017. The modular structure of pK1134-KPC was divided into the backbone and two separated accessory regions, including a *bla*_KPC-2_ region. The *bla*_KPC-2_ region (Figure 2) was 32.09 kb in length with insertion of five accessory modules: IS*5*, IS*Kpn19*, IS*Ec22*, a truncated Tn*1696*, and a truncated Tn*6296*. Tn*6296*, a 14.49-kb Tn*21* family unit transposon initially found in *K. pneumoniae* strain KP048 (Shen et al., 2009) and composed of the core backbone structure IRL (inverted repeat left)–*tnpA* (transposase)–*tnpR* (resolvase)–*res*–IRR-1 (inverted repeat right)–Δ*mcp* (truncated methyl-accepting chemotaxis protein)–IRR-2, with insertion of the local *bla*_KPC_ genetic environment, which consisted of the following: Δ*ISKpn6*, *bla*_KPC-2_, and Tn*6376*.

### Population structure and antibiotic resistance of *Klebsiella pasteurii*

**Figure 3:**
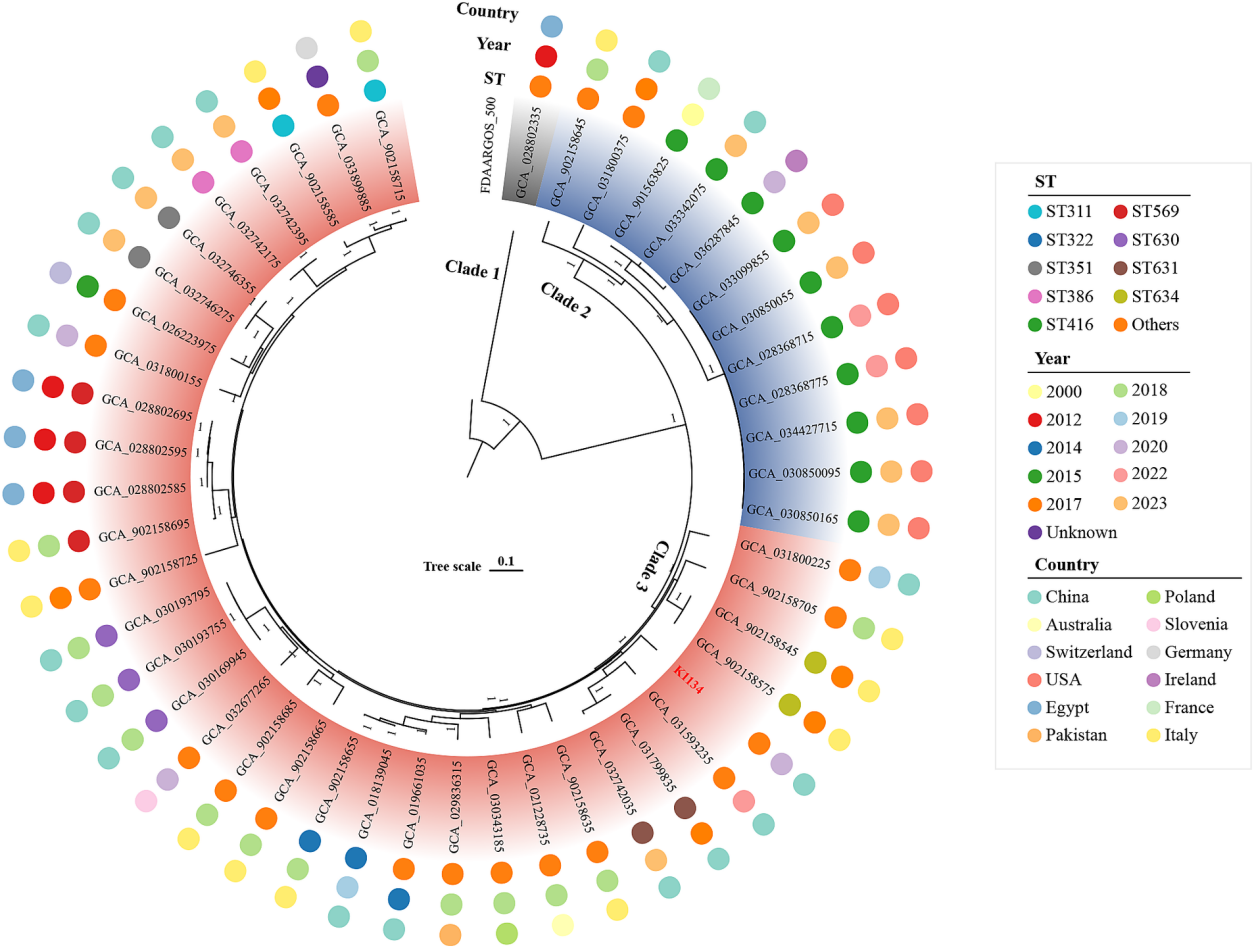
A maximum-likelihood phylogenetic tree of *K. pasteurii* isolates. Bar corresponds to scale of sequence divergence. *K. oxytoca* strain FDAARGOS_500 (accession number CP033844) is used as the outgroup. The strain K1134 is marked with red.

To further investigate the population structure and antibiotic resistance of *K. pasteurii*, we then performed the species classification and phylogenomic analysis on a collection of 48 global sequenced *K. pasteurii* isolates from 12 countries (Figure 3 and Supplementary Table S1), including the above one together with the other 47 from GenBank (last accessed July 1 2024). By comparison with the reference, all strains belonged to *K. pasteurii* based on the threshold of 95% ANI for genospecies delineation. Through genomic analysis, we identified three unique clades within the species: Clade1 (*n* = 1), Clade2 (*n* = 12) and Clade3 (*n* = 35). 35.45% (*n* = 17) of the isolates were from China (Supplementary Figure S1), and 15 isolates, including K1134 in this study, belonged to Clade3. Most of the other strains came from Western Europe, East Asia and North America (Supplementary Table S1). In addition, we performed MLST analysis on these *K. pasteurii* strains. The results showed that ST416 (*n* = 10, 20.83%) was the most common ST, and this ST type was only found in Clade2. Through MLST analysis, 10 novel STs, designated as ST628 to ST637 were identified, which had not been previously reported in the ST database, including the only strain in Clade1, whose identification result was ST629. The analysis revealed a high level of genetic diversity among the strains.

**Figure 4:**
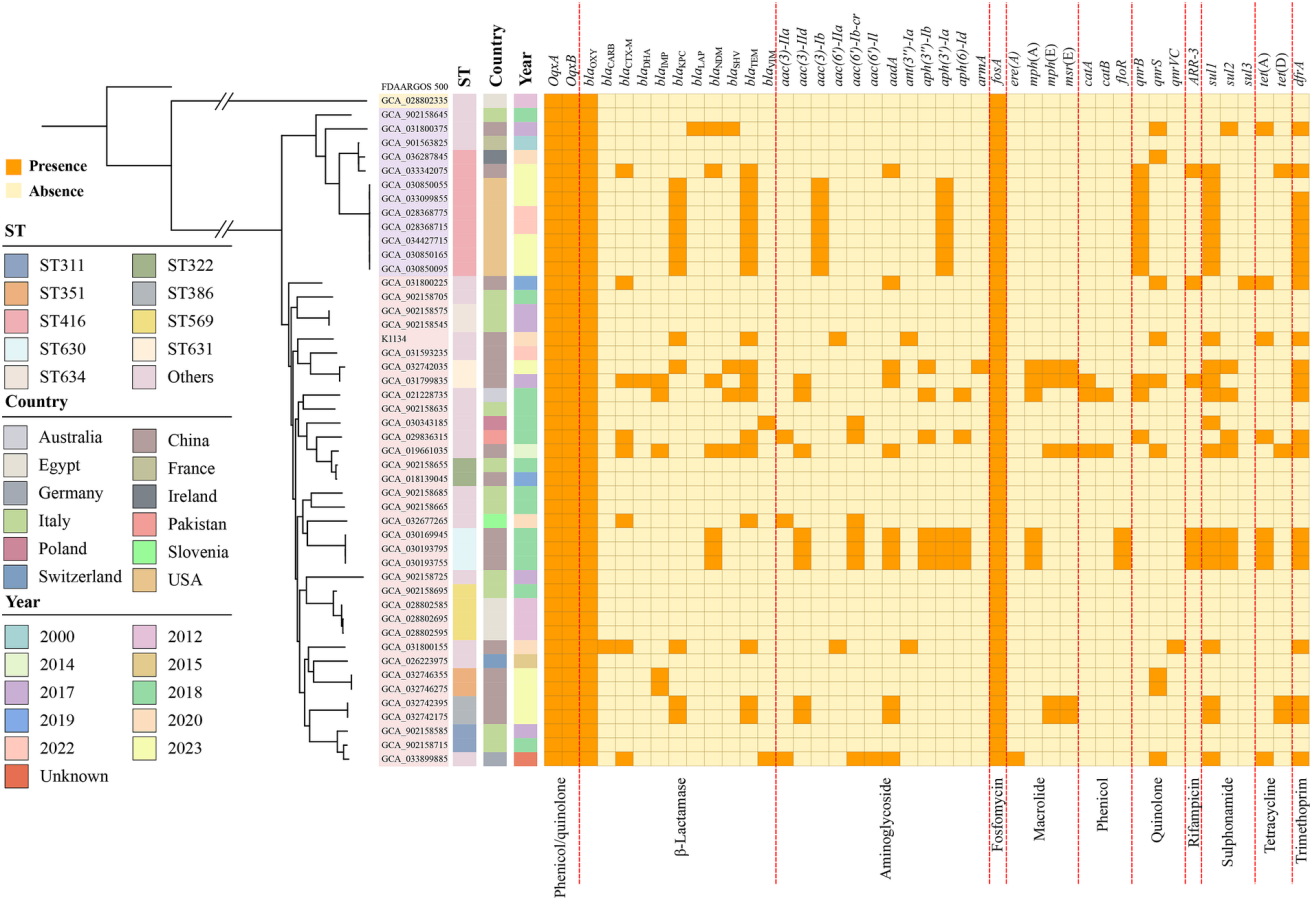
The maximum-likelihood tree and ARGs distributions in *K. pasteurii*. The matrix of ARGs is present for all *K. pasteurii* genomes against the dendrogram. The top of the matrix indicates the names of ARGs, while the bottom of that represents the groups of ARGs. The colored cells represent the “presence” of the ARGs.

At least 43 resistance genes, involved in resistance to 11 different categories of antibiotic, were identified in these 48 global *K. pasteurii* genomes (Figure 4). KoSC strains are in general intrinsically resistant to β-lactams (Dabernig-Heinz et al., 2024), in addition to *bla*_OXY_, efflux pump-encoding *oqxA*/*oqxB* and the fosfomycin resistance gene *fosA* were also found in all isolates. Of these 48 *K. pasteurii* isolates, 20 were identified as containing no other resistance genes, and the remaining 28 isolates all contained one or more acquired antimicrobial resistance genes. Other drugs, such as trimethoprim resistance gene *dfrA* (*n* = 22), sulphonamide resistance gene *sul1* (*n* = 21), and β-lactamase resistance gene *bla*_TEM_ (*n* = 18) were found in most isolates. *sul1* genes are often situated within class 1 integrons and usually co-exist in the integrons with other resistance genes, such as aminoglycoside resistance genes and quinolone resistance genes (Jacoby et al., 2014). All in all, *K. pasteurii* has a large number of acquired antimicrobial resistance genes, which can carried by diverse AGEs such as plasmids.

### Transmission of *bla*_KPC_ in *Klebsiella pasteurii*

**Figure 5:**
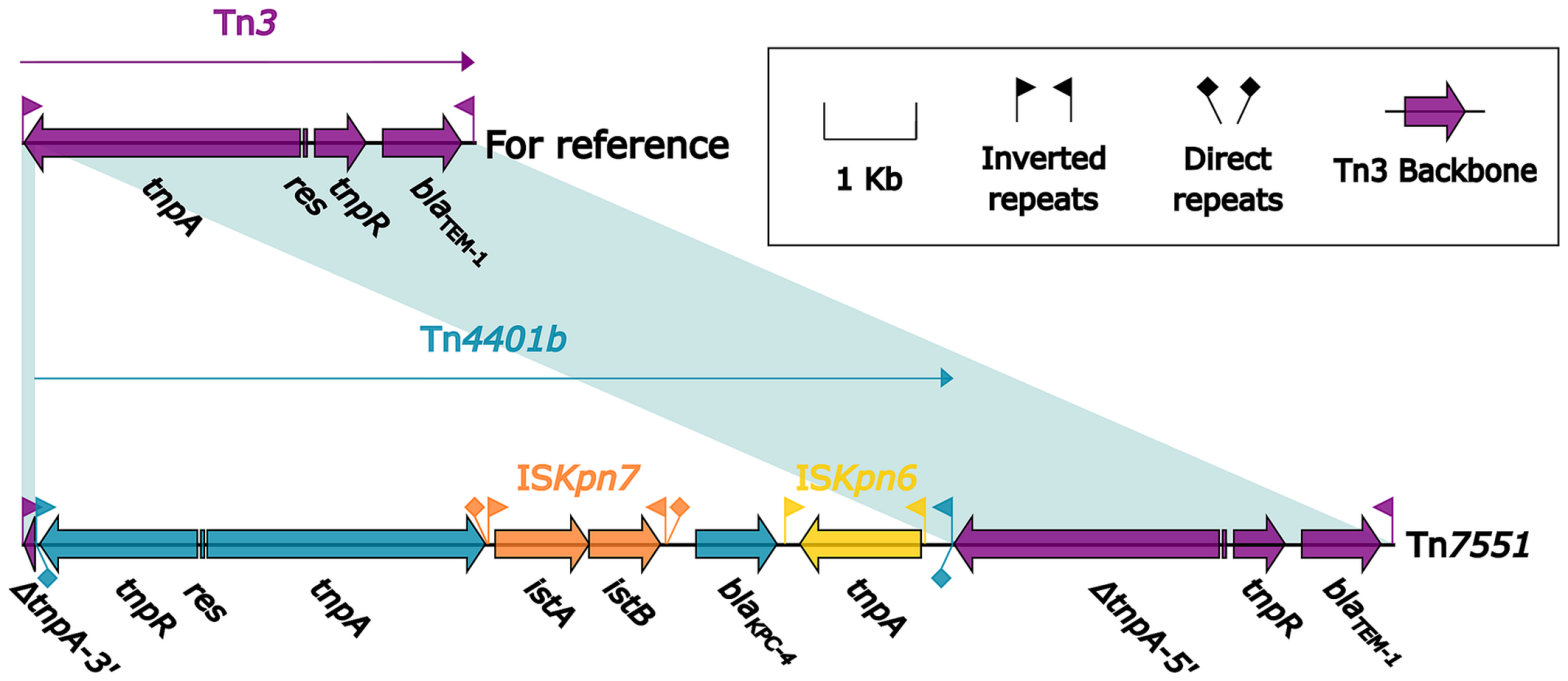
Comparison of Tn*3* and Tn*7551*. Genes are denoted by arrows. Genes, mobile elements and other features are colored based on their functional classification. Shading denotes regions of homology (nucleotide identity >95%). The accession number of Tn*3* used as reference is HM749966.

*bla*_KPC_ gene was detected in 12 isolates, and 12 *bla*_KPC_-carrying AGEs were identified from the sequences of the isolates, which belonging to two groups: Tn*7551* and Tn*6296*-related elements. Seven isolates contained the *bla*_KPC-4_ carrying Tn*7551* (Figure 5), which was a Tn*3*-family unit transposon and composed of the core backbone structure IRL–*tnpA*–*res*–*tnpR*–*bla*_TEM-1_–IRR, with a Tn*4401*-family unit transposon Tn*4401b* inserted into *tnpA*, and it had terminal 32-bp IRL/IRR pairs and were further bracketed by 5-bp direct repeats (DRs; target site duplication signals for transposition). The remaining four strains were detected to carry *bla*_KPC-202_ and their genetic environment was related to Tn*6296*. However, due to the inability to obtain the complete genome sequences, it is not possible to describe the specific genetic environment of *bla*_KPC-202_ from the four isolates. In addition, according to previous research, these genes are commonly carried by plasmids (Long et al., 2022). *repA*_IncFIB_ and *repA*_IncHI5_ were also identified from the genome sequences.

### Genes for production of the enterotoxins tilimycin and tilivalline

The gene cluster for tilimycin and tilivalline contains the regulators *npsC* and *marR*, *mfsX* (multidrug efflux MFS transporter), *uvrX* (excinuclease ABC subunit UvrA), an NRPS operon including *npsA* (amino acid adenylation domain-containing protein), *thdA* (acyl carrier protein), and *npsB* (nonribosomal peptide synthetase), and an *aroX* operon including *aroX* (3-deoxy-7-phosphoheptulonate synthase), *dhbX* (2,3-dihydro-2,3-dihydroxybenzoate dehydrogenase), *icmX* (isochorismatase), *adsX* (2-amino-2-deoxy-isochorismate synthase), and *hmoX* (4 hydroxyphenyl acetate-3-monooxygenase) (Yang et al., 2022; von Tesmar et al., 2018). Our results showed that these virulence factors were present in almost all strains (Supplementary Figure S2). The isolates with incomplete gene clusters were all derived from Clade3. Among them, one strain lacked all the genes, two strains were missing both the NRPS and *aroX* operons, and one strain exhibited a partial deletion within the *aroX* operon.

## Discussion

Infections caused by KoSC are the second most common cause of *Klebsiella* infections in humans (Stewart et al., 2022). The KoSC consists of at least nine species, including *K. grimontii*, *K. huaxiensis*, *K. michiganensis*, *K. oxytoca*, *K. pasteurii*, *K. spallanzanii*, and three unnamed novel species. Phylogroups (Ko) has been established based on differences in the intrinsic β-lactamase gene *bla*_OXY_ encoded by KoSC, and *K. pasteurii* represents the phylogroup Ko4 (Shibu et al., 2021). In this study, all *K. pasteurii* strains contained the gene *bla*_OXY-4_. Moreover, the identity of all 48 genomes included in this work was confirmed by ANI analysis, which shared 98.10–100.00% ANI with each other. Therefore, all strains were confirmed as belonging to the species *K. pasteurii*.

As shown in this study, three unique clades were identified of the species: Clade1, Clade2 and Clade3, and most of which were identified as Clade3 (72.92%), including the reference strain Sb-24, and the K1134 sequenced in this study. The only Clade1 strain was from Egypt in 2012. Geographic or temporal patterns also reveal genetic diversity within the species, and it was found that the strains with the same spatial and temporal distribution also had genomic differences. The MLST is a powerful tool for epidemiological investigation of bacterial pathogens. A total of 28 distinct STs were identified, including 10 that were novel to this study. ST416 (20.83%) was the most common ST in these strains, and this ST type was only found in Clade2 and has become prevalent worldwide. Except for ST416, ST569 and ST630, which were slightly common (*n* ≥ 3), other STs were mostly detected in a single strain.

Acquired antimicrobial resistance is an emerging concern for *K. pasteurii*. Whole-genome sequencing revealed K1134 includes a *bla*_KPC-2_-carrying plasmid pK1134-KPC, which belongs to the IncFII_pCP020359_ group. Comparative genomic analysis showed that Tn*6296* is associated with the local *bla*_KPC_ genetic environment. *bla*_KPC_ gene was detected in other 11 strains, and the 12 *bla*_KPC_-carrying AGEs belong to two groups: Tn*7551* and Tn*6296*-related elements. The seven strains carrying Tn*7551* transposons showed high homology and were isolated from the United States in 2022 and 2023 with ST type ST416, suggesting clonal spread of these strains. The strains carrying Tn*6296*-related elements exhibit a dispersed distribution across the phylogenetic tree, indicating a lack of clear clonal clustering. This suggests substantial genetic diversity within the group of drug-resistant strains.

In the 48 global *K. pasteurii* genomes, at least 43 resistance genes were identified, conferring resistance to 11 classes of antibiotics and heavy metals, with strains generally being intrinsically resistant to β-lactams. These genomes also harbored a variety of acquired resistance genes. KoSC carries intrinsic β-lactamase-encoding *bla*_OXY_ and fosfomycin resistance gene *fosA* (Merla et al., 2019). Many resistance genes in addition to these co-exist in these starins, suggest that these strains are multidrug-resistant, with the ability to resist a wide range of antibiotics. In addition, coexistence of two carbapenem genes was found in several strains, which will further complicates the treatment of *K. pasteurii* infections. Analysis of KoSC collected at a hospital in Australia showed that one *K. pasteurii* strain carried *bla*_IMP-4_ IncL/M plasmids, which indicates that resistance plasmids similar to those found in other Enterobacterales, were found in KoSC, so it is possible for KoSC to develop multidrug resistance (Stewart et al., 2022).

KoSC is rising as a significant opportunistic pathogen causing nosocomial infections (Neog et al., 2021). The pathogenicity of the microbe has been attributed to the production of cytotoxins, namely tilivalline and tilimycin, which are implicated in certain intestinal disorders (Minami et al., 1992). Our results showed that the related virulence factors were present in almost all *K. pasteurii* strains, which raises concerns about the widespread potential for these strains to cause intestinal infections in hospital settings, particularly in high-risk areas such as intensive care units and neonatal care units.

## Conclusion

This study reveals the molecular epidemiology, antimicrobial resistance profiles, and pathogenicity of *K. pasteurii*. First, *K. pasteurii* is part of the KoSC and is classified into three major clades, with Clade3 being the most common. MLST analysis identified 28 distinct STs, with ST416 being the most prevalent and widely distributed globally. Additionally, *K. pasteurii* exhibits significant antimicrobial resistance, particularly to β-lactams. Various resistance genes including *bla*_KPC_ were identified in these strains, further complicating treatment options. The study underscores the role of plasmids and other genetic elements in conferring resistance. The presence of plasmids and other genetic elements, such as transposons plays a crucial role in the emergence of multidrug-resistant *K. pasteurii* strains. This underscores the need for careful monitoring and control measures in healthcare settings. Almost all *K. pasteurii* strains in the study carried these virulence factors related to tilivalline and tilimycin, raising concerns about their potential to cause infections in hospital settings. In conclusion, *K. pasteurii* is an emerging opportunistic pathogen with broad antimicrobial resistance and significant virulence potential. These factors warrant increased clinical attention to manage its spread and infection risk, particularly in hospital environments.

## Data Availability

The datasets presented in this study can be found in online repositories. The names of the repository/repositories and accession number(s) can be found in the article/Supplementary material.

## Footnotes

^1^ https://github.com/ruanjue/smartdenovo

^2^ https://www.bioinformatics.babraham.ac.uk/projects/fastqc

^3^ https://rast.nmpdr.org/

^4^ https://web.expasy.org/docs/swiss-prot_guideline.html

^5^ https://www.ncbi.nlm.nih.gov/refseq/

^6^ https://card.mcmaster.ca/browse

^7^ https://cge.cbs.dtu.dk/services/ResFinder/

^8^ https://www-is.biotoul.fr/

^9^ http://integrall.bio.ua.pt/?glossary

^10^ https://www.ucl.ac.uk/eastman/tn-number-registry

^11^ https://inkscape.org/en/

^12^ https://mummer.sourceforge.net/

^13^ http://www.repeatmasker.org/
