# Supplementary figures and images for: Unraveling the genomic epidemiology and plasmid-mediated carbapenem resistance of Klebsiella pasteurii
Source: Front Microbiol. 2025 Mar 17;16:1561624. doi: 10.3389/fmicb.2025.1561624 (PMC11955625; doi:10.3389/fmicb.2025.1561624)

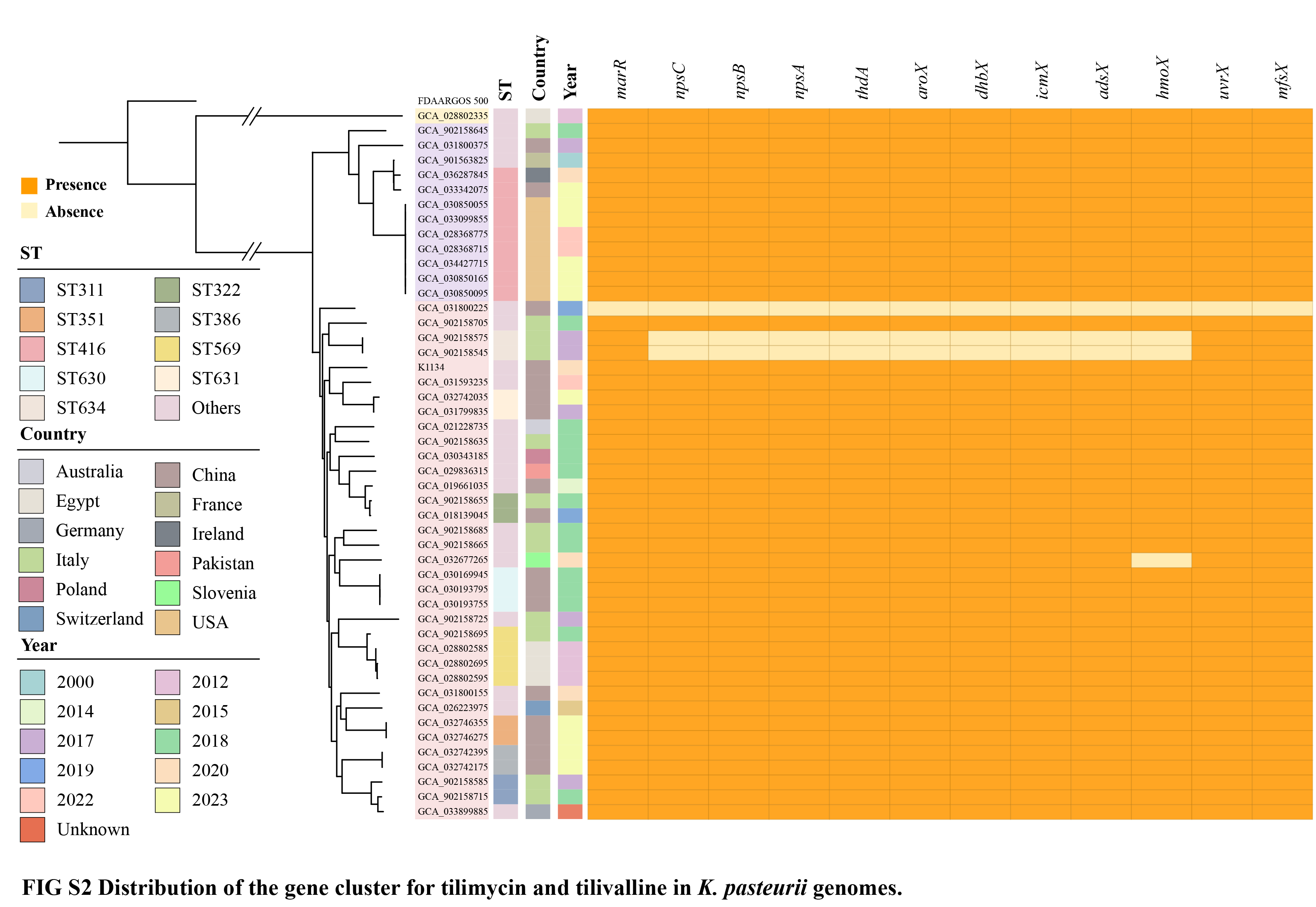

Supplement: Supplementary file 2 [file Image_2.tif]
